# Privately vertically mining of sequential patterns based on differential privacy with high efficiency and utility

**DOI:** 10.1038/s41598-023-43030-z

**Published:** 2023-10-19

**Authors:** Wenjuan Liang, Wenke Zhang, Songtao Liang, Caihong Yuan

**Affiliations:** 1 College of Computer and Information Engineering, Henan University, Kaifeng, 475004 China; 2 Henan Engineering Research Center of Intelligent Technology and Application, Henan University, Kaifeng, 475004 China; 3 Vedio Cloud Research Center, Bilibili Inc, Shanghai, 200433 China

**Keywords:** Mathematics and computing, Computer science

## Abstract

Sequential pattern mining is one of the fundamental tools for many important data analysis tasks, such as web browsing behavior analysis. Based on frequent patterns, decision-makers can obtain both economic gains and social values. Sequential data, on the other hand, frequently contain sensitive information, and directly analyzing these data will raise user concerns from a privacy perspective. Differential privacy (DP), as the most popular privacy model, has been employed to address this privacy concern. Most existing DP-Solutions are designed to combine horizontal sequence pattern mining algorithms with differential privacy. Due to the inefficiency of horizontal algorithms, their DP-Solutions cannot ensure high efficiency and accuracy while offering a high privacy guarantee. Therefore, we proposed privVertical, a new private sequence pattern mining scheme combining the vertical mining algorithm with differential privacy to achieve the above objective. Unlike DP-solutions based on horizontal algorithms, privVertical can promote efficiency by avoiding performing costly database scans or costly projection database constructions. Moreover, to promote accuracy, a differentially private hash MapList (called privHashMap) is designed to record frequent concurrency items and their noisy support based on the Sparse Vector Technique. PrivHashMap is used to pre-pruning excessive infrequent candidate sequences in private mining, and Sparse Vector Technique is used to promote the accuracy of PrivHashMap. After pruning these invalid candidate sequences, less noise is required to achieve the same level of privacy, increasing the accuracy of private mining. Theoretical privacy analysis proves privVertical satisfies $\varepsilon$-differential privacy. Experiments show that privVertical achieves higher accuracy and efficiency while achieving the same privacy level.

## Introduction

**Figure 1:**
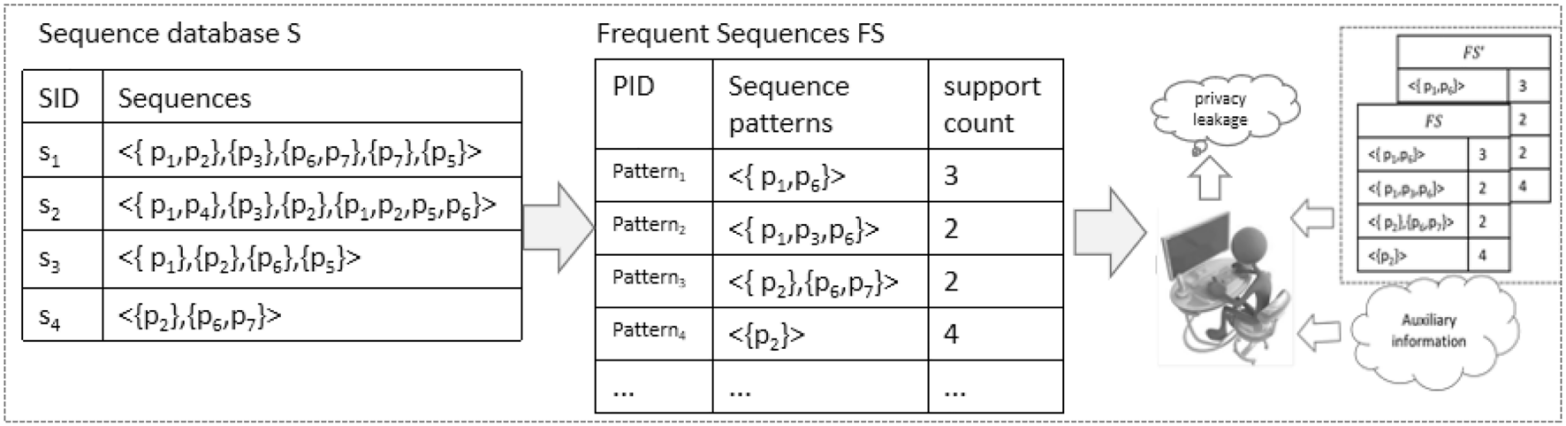
Example of sequence pattern mining and privacy leakage.

Sequential pattern mining (SPM) is one of the fundamental tools for many important data analysis tasks, such as web browsing behavior analysis. It is to find sequential patterns whose support is no less than a specified threshold^1^. Data analysts can make a more accurate prediction by analyzing frequent sequential patterns. Since sequential data often contains sensitive information, directly mining frequent patterns will raise user concerns from a privacy perspective. As shown in Fig. 1, given a sequence database S, and suppose the support threshold is 50%, we can get the frequent sequence patterns by executing the SPM algorithm. By observing the output of frequent sequential patterns and their frequency, the attackers can derive sensitive data of individual users based on background knowledge, and thus privacy leakage may occur. Therefore, it is crucial for sequence data analysis to understand how to protect privacy in sequence pattern mining.

Early research frequently used cryptography or k-anonymity techniques to address the privacy problem in SPM^2–5^. However, it has shown that they are vulnerable to many privacy attacks and can not provide sufficient protection, including background and combination attacks. Differential privacy^6^, as the most popular data privacy model, has recently been paid close attention to by researchers and industrial communities. Differential privacy can provide a stronger privacy guarantee than early privacy models. By perturbing the sequence pattern mining algorithm with random noises according to differential privacy, even if the attacker acquires all background knowledge except the attack target, he cannot infer the sensitive information of individual users^7^.

Recently, there have been several differentially private sequential pattern mining solutions^8–17^. Most of them are designed based on horizontal algorithms (e.g. Apriori^18^, PerfixSpan^19^). For example, Bonomi et al.^8^ proposed a differentially private mining scheme based on the PerfixSpan algorithm. They first constructed a model-based prefix tree to mine prefixes and the candidate set of substring patterns. Then they refined the frequency of the substring patterns in the successive phase to reduce the perturbation noise. Xu et al.^9, 10^ proposed a differentially private SPM scheme based on Apriori-based algorithms. They perturbed their frequencies with random noise to satisfy differential privacy. To promote the accuracy of private mining, they proposed shrinking long sequences and filtering invalid candidate patterns based on a sampling database. These DP-Solutions can not provide high efficiency and accuracy while providing a high level of privacy. Reasons are summarized as follows: (1) Low efficiency: horizontal algorithms contain too many database scans or prefix-projected database construction. Private processing needs to be designed for each scan or construction, which reduces efficiency. (2) Low accuracy: Lower accuracy is caused by two factors: the first one is that more candidates generated in private mining result in lower accuracy because the amount of noise must be proportionate to the number of candidates. The other is that refined or sampling errors exist in calculating the frequency of patterns after perturbing, which lowers the accuracy. A practical and available mining scheme should provide a high privacy level while ensuring its efficiency and accuracy, and existing solutions can not achieve the above objectives at the same time.

Therefore, we attempt to design a differentially private sequence pattern mining scheme with both high efficiency and utility, while providing a high level of privacy. As far as we know, there are two types of non-private SPM algorithms: horizontal mining algorithms^18–21^ and vertical mining algorithms^22–26^.The former works are characterized by performing costly database scans or projection database constructions. The latter works are characterized by scanning the original database to create its vertical format (called IDList) and then generating candidate patterns through the cross-join of IDLists. Vertical mining algorithms are more effective than horizontal mining algorithms. In light of the advantages of the vertical mining algorithm, we attempt to design a private mining scheme based on the vertical sequential pattern mining algorithm. To make the private mining satisfy differential privacy constraints, we designed a random noise addition scheme combined with the vertical mining process. To further improve the accuracy, we designed a differentially private hash map list (called as privHashMap) to record frequent co-currency items based on the Sparse Vector Technique^2^. PrivHashMap is used to pre-pruning excessive invalid candidate sequences in private mining. After pruning these invalid candidate sequences, less noise is needed to maintain the same level of privacy, improving the accuracy.

## Related works

### Differentially private SPM

Sequential pattern mining provides knowledge, and at the same time, it has the risk of privacy disclosure. Several differentially private sequence pattern mining schemes (DP-SPM) have been proposed to address the above privacy concern. Bonomi et. al.^8^ first proposed a differentially private mining scheme based on the PerfixSpan algorithm. They first constructed a model-based prefix tree to mine prefixes and a candidate set of substring patterns. Then they refined the frequency of the substring patterns in the successive phase to reduce the perturbation noise. Xu et. al.^9, 10^ proposed a differentially private SPM scheme based on the Apriori-based algorithm. They first designed a sequence shrinkage technique to reduce the length of the sequence. Then they used the statistical information of the sampling data set to prune invalid candidate patterns to improve the accuracy. In contrast to the above DP-SPM methods, the following works concentrate on differentially private SPM under different constraints. Cheng et. al.^11^ proposed a private mining scheme DP-MFSM for maximum frequent sequence mining. Li et al.^12^ proposed a differentially private sequence pattern mining algorithm with time constraints. Le et. al.^13^ proposed a differentially private sequential pattern mining scheme considering time intervals for electronic medical record systems. Supposed data managers are not trusted, Le et al.^15^ and Afrose et al.^16^ employed local differential privacy to protect the privacy in sequential pattern mining. Wang et al.^14, 17^ proposed several privacy-preserving schemes for critical or top-k patterns mining over data streams.

### Non-private SPM

Non-private SPM algorithms are divided into two categories:mining algorithms based on horizontal database format and mining algorithms based on vertical database format. (1) SPM algorithms based on horizontal database format. Apriori-based algorithms (e.g. AprioriAll^18^) are representative horizontal mining algorithms. In Apriori-based algorithms, candidate patterns are generated according to downward closure property, and the original dataset is scanned several times to calculate the support of candidate patterns. They are inefficient due to multiple database scans and large candidate patterns. Some improved algorithms^19–21^ are proposed to improve efficiency, such as PrefixSpan^19^. PrefixSpan explores prefix projection to reduce the efforts of candidate subsequence generation and also belongs to horizontal algorithms. However, efficiency is also its bottleneck due to multiple constructions of prefix-projected databases. (2) SPM algorithms based on vertical database format. Vertical mining algorithms are proposed to solve the problem of low mining efficiency caused by multiple data scans. Spade^22^ is a representative vertical mining algorithm. The efficiency of vertical algorithms can be improved by eliminating database scans. However, too many cross-connection operations of vertical lists also result in lower mining efficiency. The works^23–26^ proposed several improvement strategies to address this issue.

## Preliminaries

### Differential privacy

#### Definition 1

(*Differential privacy*)^2^. If the output of a randomization algorithm *M* on any neighboring sequence datasets *S*, $S'$ satisfies the following constraints:

1 $$\begin{aligned} e^{-\varepsilon } \le \frac{Pr[M(S)\in FS]}{Pr[M(S')\in FS]} \le e^{\varepsilon } \end{aligned}$$

*M* is said to be $\varepsilon$-differentially private. *FS* is an arbitrary subset of the output domain of *M*. $\varepsilon$ is called as privacy budget. It is used to control the privacy level of *M*. A smaller $\varepsilon$ represents a stronger privacy protection. *S* and $S'$ represent neighboring sequential datasets, which means that $|S|-|S'|=1$.

#### Definition 2

(*Sensitivity*)^2^. Let $\Delta f$ denote the sensitivity of the query function *f*, and it can be calculated as follows:

2 $$\begin{aligned} \Delta f = max_{S,S'}\parallel f(S)-f(S')\parallel _1 \end{aligned}$$

where $\Delta f$ equals to the maximum $L_1$ norm distance between *S* and $S'$. The sub-index 1 represents $L_1$ norm, which means the sum of the magnitudes of the vectors in space.

#### Definition 3

(*Laplace mechanism*) Let *Q* denote a query function sequence with dimension *n*, $\Delta Q$ denotes the sensitivity of *Q*. Let $\xi =<\xi _1, \xi _2,\ldots , \xi _n>$ be a random noise vector, $\xi _i=Lap(\lambda )$, $\lambda =\Delta Q/\varepsilon$, and the probability density function is $p(x/\lambda )= 1/2\lambda \cdot exp(-|x|/\lambda )$. If add $\xi$ to *Q*(*S*), that is

3 $$\begin{aligned} M(S) = Q(S) + <\xi _1, \xi _2,\ldots , \xi _n> \end{aligned}$$

*M* is said to satisfy $\varepsilon$-differential privacy.

#### Theorem 1

(Sequential Composition)^2^. Let $M_1, \cdots , M_n$ be m randomized algorithms, $M_i$ provides $\varepsilon _i-$differential privacy $(1\le i\le n)$, and a sequence of $M_i(S)$ provides $\sum {\varepsilon _i}$-differential privacy.

### Vertical sequence pattern mining

#### Sequence database

Let $U=\{p_1, p_2,\ldots , p_l\}$ denote the universe set of items, and $p_i$ represents a single item. Let $I_{x}=\{p_i, p_j,\ldots , p_k\}\subseteq U$ denote an unordered set of distinct items. A sequence $s=<I_1, I_2,\ldots , I_n>$ is an ordered arrangement of itemsets such that $I_k \subseteq U (1\le k \le n)$. As shown in Fig. 1, the sequence database *S* consists of a set of sequences $\{s_1, s_2,\ldots , s_n\}$, and the first sequence $s_1$ contains five itemsets $<\{p_1, p_2\},\{p_3\},\{p_6, p_7\},\{p_7\}, \{p_5\}>$.

#### Frequent sequence pattern

If the support of a sequence pattern $pattern_i$ exceeds a certain threshold, then $pattern_i$ is a frequent sequence pattern. A frequent pattern with length *k* is called a frequent *k*-sequence pattern.

#### Vertical sequence database format^24^

**Figure 2:**
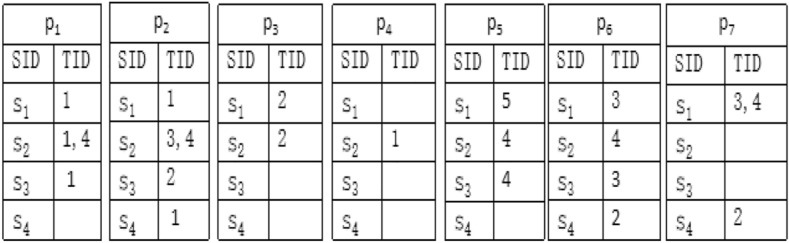
Vertical format of sequence database (IDLists of single items).

In the vertical database format, each entry represents an item and indicates the list of sequences where the item appears (SID) and the timestamps when it appears (TID). A structure named IDList is associated with each pattern. The support of a larger pattern can be quickly calculated by performing join operations with IDLists of smaller patterns. In the vertical mining process, IDLists of single items are created by scanning the database once. Then IDLists of larger patterns can subsequently be obtained by conducting join operations on IDLists of smaller patterns. Figure 2. shows an example of vertical database format according to the sequence database in Fig. 1. Taking the construction of IDList of $p_1$ as an example, $s_1=<\{p_1, p_2\},\{p_3\},\{p_6, p_7\},\{p_7\}, \{p_5\}>$, TID that $p_1$ appears in $s_1$ is $\{1\}$. Therefore, the first entry of its IDList is $(s_1,\{1\})$. $s_2=<\{p_1, p_4\},\{p_3\},\{p_2\},\{p_1, p_2, p_5, p_6\}>$, TID that $p_1$ appears in $s_2$ is $\{1,4\}$. Therefore, the second entry of its IDList is $(s_2,\{1,4\})$. $s_3=<\{p_1\},\{p_2\},\{p_6\},\{p_5\}>$, TID that $p_1$ appears in $s_3$ is $\{1\}$. Therefore, the third entry of its IDList is $(s_3,\{1\})$. $s_4=<\{p_2\},\{p_6,p_7\}>$, TID that $p_1$ appears in $s_4$ is none. Therefore, the fourth entry of its IDList is $(s_4,\{\} )$.

## The basic idea of the algorithm

The sequence patterns mining algorithm must be randomized to guarantee data privacy. Our scheme is designed based on the vertical sequence pattern mining algorithm^24^ to achieve high efficiency. Differential privacy is employed to perturb the vertical mining algorithm. To make the mining process satisfy $\varepsilon$-differential privacy, a straight solution is designed as follows: (1) Perturb the IDLists of candidate patterns according to the Laplace mechanism. (2) Based on the perturbed IDLists, calculate noisy supports of candidate patterns by performing the cross-join operations of noisy IDLists. (3) Filter out frequent patterns based on their noisy supports and the minimum support threshold.

In the above process, the noise required for perturbation should be proportional to the sensitivity of the mining process and inversely correlated with the privacy budget. Suppose the maximum cardinality of frequent sequences is *m*, the privacy budget $\varepsilon$ is divided equally in the iterative mining process, and thus the budget allocated in each iteration is $\varepsilon /m$. Suppose $Q=\{q_1, q_2,\ldots , q_n\}$ is the query function in the private mining, where $q_i$ represents the sub-query of the number of candidate *i*-sequences in the *i*th iteration. The sensitivity of $q_i$ equals to the maximum number of candidate *i*-sequences $|CS_i|$. Therefore, add $Lap(\varepsilon /m\cdot |CS_i|)$ noise to the IDLists of candidate *i*-sequences, the *i*th iteration process can satisfy $\varepsilon /m$-differential privacy. After *m* iterations, it can easily prove that the private mining process satisfies $\varepsilon$-differential privacy. However, the sensitivity of the straight solution is too high, which results in a large amount of noise required for privacy protection.

PrivVertical, an improved algorithm, is proposed to reduce high sensitivity. PrivVertical consists of three components: (1) Construction of privHashMap based on sparse vector technology. (2) Perturbation of IDLists. (3) Infrequent candidates pruning based on privHashMap. To satisfy $\varepsilon$-differential privacy, the privacy budget can be allocated as follows: the budget for the construction of privHashMap is $\varepsilon _1=\alpha \cdot \varepsilon$, and the budget for the remaining two components is $\varepsilon _2=(1-\alpha ) \cdot \varepsilon$, where $0 \le \alpha \le 1$.

**(1) Construction of privHashMap based on sparse vector technology**

**Figure 3:**
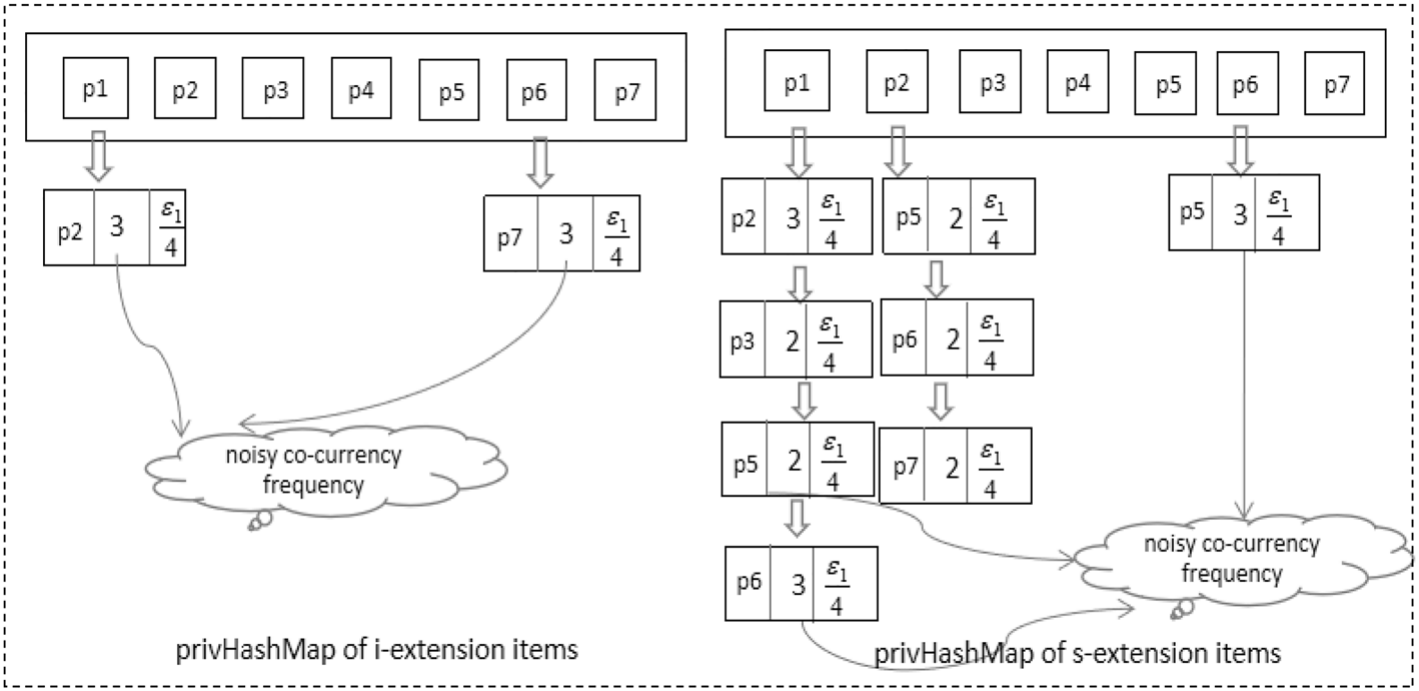
Example of privHashMap.

There are too many infrequent candidates generated in private mining. The existence of these patterns won’t affect the accuracy of non-private mining, but it will lower the accuracy of private mining. Infrequent candidates should be pruned as early as possible to address this issue, and PrivHashMap is proposed for this purpose. In the vertical mining process, when generating candidate *i*-patterns based on frequent $(i-1)$-patterns, the generation style can be divided into two types: one is *i*-extension, and the other is *s*-extension. Suppose $s=\{I_1, I_2,\ldots , I_n\}$ is a sequence, $I_k \subseteq U$. If $p_j,p_k \in I_x$, for an integer *x* such that $1\le x \le n$ and $p_k \succ _{lex} p_j$, the item $p_k$ is said to succeed by *i*-extension. Otherwise, if $p_j\in I_x$ and $p_k \in I_y$ for some integers *x* and *y* such that $1\le x < y \le k$, the item $p_k$ is said to succeed by *s*-extension. According to the sequence database in Fig. 1, all single items and their extension items can be seen in Fig. 3. For example, $\{p_2\}$ is an *i*-extension item of $p_1$, and $\{p_2, p_3, p_5, p_6\}$ are *s*-extension items of $p_1$. PrivHashMap records extension items and their co-occurrence frequencies of each single item. They are used to pre-prune infrequent candidates in the iterative mining process. After pruning infrequent candidates, sensitivity can be reduced, the required noise can be lower, and thus accuracy can be improved. In our implementation, if the co-occurrence frequency information is recorded in an $n\times n$ matrix, there will be a large waste of empty entries. The existence of these empty entries will seriously affect the candidate pattern filtering efficiency. So we implemented the co-occurrence frequency table as a hash table of HashSets. Each HashSet corresponds to an item $p_k$ and its extensive frequent co-occurrence items. Figure 3 shows an example of privHashMap.

PrivHashMap is constructed based on original sequence data. To avoid possible privacy leakage, its construction should be perturbed by Laplace noise. In this process, the sparse vector technique (SVT)^2^ is employed to ensure accuracy while providing a high privacy level. The private construction can be implemented in two steps: Firstly, perturb the support threshold $\sigma$ with Laplace noise $Lap(2/\varepsilon _1)$, and get a noisy threshold $\hat{\sigma }$. Secondly, perturb the frequency of each candidate 2-sequence $C(r_2)$ with Laplace noise $Lap(4\cdot \Delta /\varepsilon _1)$. Compare $C(r_2)+Lap(4\cdot \Delta /\varepsilon _1)$ with noisy support $\hat{\sigma }$. Output ’above the threshold’ if $C(r_2)+Lap(4\cdot \Delta /\varepsilon _1)\ge \hat{\sigma }$, otherwise output ’below the threshold’. Here $\Delta$ represents the sensitivity of the private construction, and its value equals the number of frequent 2-sequences $|FS_2|$. In this way, PrivHashMap is constructed with both high accuracy and a high level of privacy.

### Theorem 3

The construction of privHashMap satisfies $\varepsilon _1$-differential privacy.

### Proof

Let *A* represent the query function of the frequency of co-currency items, and $A_i$ represents the query function of the *i*th co-currency items. For any neighboring sequence datasets *S*,$S'$, if we can prove $Pr[A(S)\in O]\le e^{\varepsilon }\cdot Pr[A(S')\in O]$, we can get algorithm 1 satisfies $\varepsilon _1$-differential privacy. Since *S* and $S'$ are neighboring sequence databases, we can get that $A_i(S')-1 \le A_i(S)\le A_i(S')+1$. Let $r=\{r_1,r_2,\ldots ,r_l\}$ represent an output vector in this process, $r_{\ge \hat{\sigma }}=\{i:r_i\ge \hat{\sigma }\}$, $r_{< \hat{\sigma }}=\{i:r_i< \hat{\sigma }\}$. Let $\rho$ represent the query function of the number of co-currency items, $Pr[\rho (S)=\Delta ]\le e^{\varepsilon _1/2}\cdot Pr[\rho (S')=\Delta ]$. Thus,

$$\begin{aligned} & Pr[A(S)\in O] \\ & =\int _{-\infty }^{+\infty }\int _{-\infty }^{+\infty }Pr[\rho (S)=\Delta ]Pr[\hat{\sigma }=z]\prod \limits _{r_i < \hat{\sigma }}{Pr[A_i(S)+l_{noise} < z]}\prod \limits _{r_i\ge \hat{\sigma }}{Pr[A_i(S)+l_{noise}\ge z]}dzd\Delta \\ &=\int _{-\infty }^{+\infty }Pr[\rho (S)=\Delta ][\int _{-\infty }^{+\infty }Pr[\hat{\sigma }=z-1]\prod \limits _{r_i < \hat{\sigma }}{Pr[A_i(S)+l_{noise} < z-1]}\prod \limits _{r_i\ge \hat{\sigma }}{Pr[A_i(S)+l_{noise}\ge z-1]}dz]d\Delta \\ &\le \int _{-\infty }^{+\infty }Pr[\rho (S)=\Delta ][\int _{-\infty }^{+\infty }e^{\frac{\varepsilon _1}{2}}Pr[\hat{\sigma }=z]\prod \limits _{r_i < \hat{\sigma }}Pr[A_i(S^{\prime})-1+l_{noise} < z-1]\prod \limits _{r_i\ge \hat{\sigma }}{Pr[A_i(S)+l_{noise}\ge z-1]}dz]d\Delta \\ &\le \int _{-\infty }^{+\infty }Pr[\rho (S)=\Delta ][\int _{-\infty }^{+\infty }e^{\frac{\varepsilon _1}{4}}Pr[\hat{\sigma }=z]\prod \limits _{r_i < \hat{\sigma }}Pr[A_i(S^{\prime})+l_{noise} < z-1]\prod \limits _{r_i\ge \hat{\sigma }}e^{\frac{\varepsilon _1}{4\Delta }}{Pr[A_i(S^{\prime})+l_{noise}\ge z]}dz]d\Delta \\ {} &\le \int _{-\infty }^{+\infty }e^{\frac{\varepsilon _1}{2}}Pr[\rho (S)=\Delta ][e^{\frac{\varepsilon _1}{4}+\Delta \cdot \frac{\varepsilon _1}{4\Delta } }[\int _{-\infty }^{+\infty }Pr[\hat{\sigma }=z]\prod \limits _{r_i < \hat{\sigma }}Pr[A_i(S^{\prime})+l_{noise} < z]\prod \limits _{r_i\ge \hat{\sigma }}{Pr[A_i(S^{\prime})+l_{noise}\ge z]}dz]]d\Delta \\ &=e^{\frac{\varepsilon _1}{2}+e^{\frac{\varepsilon _1}{4}+\Delta \cdot \frac{\varepsilon _1}{4\Delta } }}Pr[\rho (S)=\Delta ][\int _{-\infty }^{+\infty }Pr[\hat{\sigma }=z]\prod \limits _{r_i < \hat{\sigma }}Pr[A_i(S^{\prime})+l_{noise} < z]\prod \limits _{r_i\ge \hat{\sigma }}{Pr[A_i(S^{\prime})+l_{noise}\ge z]}dz]d\Delta \\ &=e^{\varepsilon _1}Pr[A(S^{\prime})\in O] \end{aligned}$$

**(2) Perturbation of IDList**

**Figure 4:**
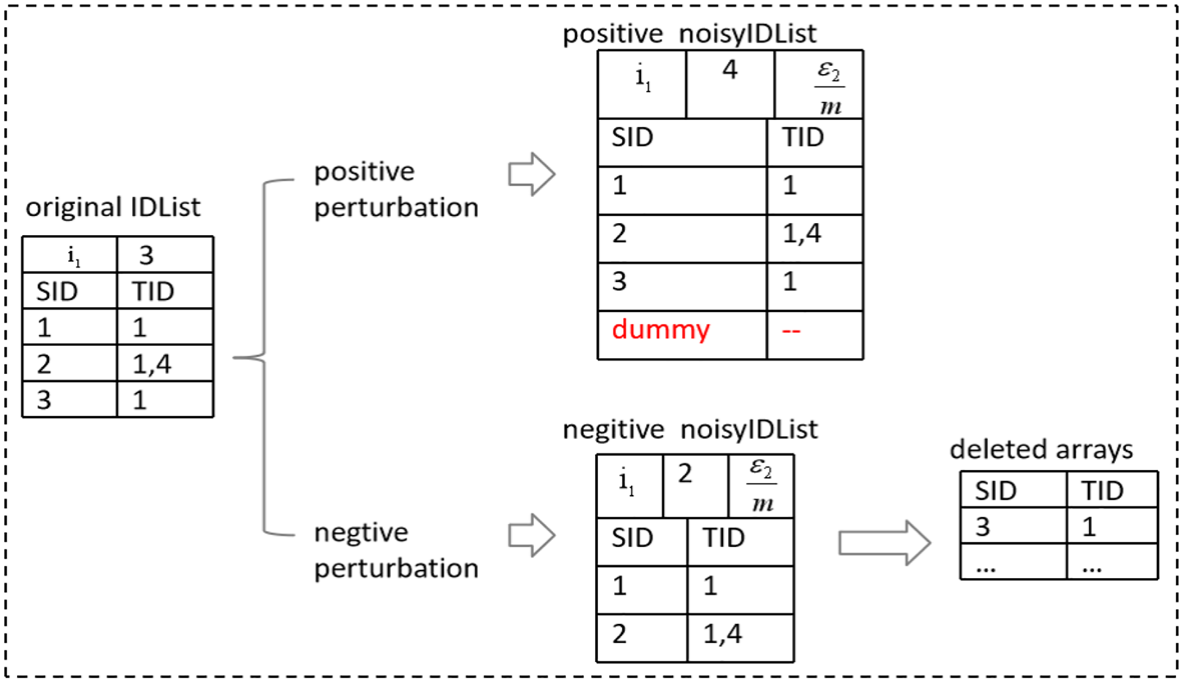
Example of IDList Perturbation.

In vertical mining, candidate *k*-patterns ($k>1$) are generated by performing the cross-join with IDLists of frequent $(k-1)$-patterns. To satisfy differential privacy, IDLists should be perturbed by random noise for privacy protection. As shown in Fig. 4, the IDList of a pattern *r* consists of a header node and several entries. The header node records the number of entries and the budget allocated here. Each entry represents the list of sequences where *r* appears (SID) and the timestamps when it appears (TID). The perturbation of the IDList follows the following two steps: First, Perturb the true entry number with the allocated budget. Second, perform consistency adjustment of IDList according to the noisy number. In step 1, for each candidate *k*-sequence, the magnitude of noise required for the perturbation of its IDList is $Lap(m\cdot \Delta f_k/n\varepsilon _2)$, where *m* represents the maximum cardinality of frequent patterns, $\Delta f_k$ represents the sensitivity, and *n* is the size of the sequence database. In step 2, consistency adjustment can be performed as follows: the perturbation of IDList can be divided into two types: positive perturbation and negative perturbation. From Fig. 4, we can see that dummy entries can be inserted into the noisyIDList when the perturbation is positive. Each dummy entry is composed of a SID value and a TID value. To ensure mining accuracy, the SID value of the dummy entry should be different from all existing SID values. In this way, the accuracy can not be affected when generating candidate patterns by performing the cross-join between IDLists. When the perturbation is negative, consistency adjustment can be implemented by deleting several entries of the IDLlist according to the noisy count.

**(3) Candidates Pruning based on privHashMap**

Vertical mining is an iteration process. In the *k*th recursive process, candidate *k*-sequences are generated by performing the cross-join of noisyIDLists of frequent $(k-1)$-sequences. Meanwhile, infrequent candidate patterns are pruned based on privHashMap. The pruning rules are as follows: Let $A_i$ and $A_j$ represent any two frequent $(k-1)$-sequence patterns, $A_i = P \cup x$, $A_j = P \cup y$, *P* is the common prefix of $A_i$ and $A_j$, *y* is the extension item of $A_ i$, *x* is the last item of $A_i$, *a* is the last item of *P*, $r = A_i \cup y$. If *y* is an *i*-extension item or *s*-extension item of *a* in privHashMap, *r* should be retained in the candidate *k*-patterns set. Otherwise, *r* should be filtered out.

For each candidate *k*-pattern *r* retained in the candidate set, perturb its IDList. The magnitude of noise for perturbation is $Lap(m\cdot \Delta f_k/n\varepsilon _2)$. Based on the noisyIDList of candidate *k*-patterns and the support threshold, frequent *k*-patterns can be filtered out. The sensitivity of the queries in the *k*th recursive mining is $\Delta f_k = min(C_{l}^{k},T_k)-|del_k|$. The analysis is as follows: The private vertical mining is a recursive process. Let $T_k$ represent the candidate *k*-sequences generated in the *k*th recursive process, frequent *k*-patterns can be filtered out from $T_k$. According to differential privacy, the magnitude of noise should be proportional to the sensitivity and inversely proportional to the budget. Suppose the query function in the private mining is $Q =\{q_1, q_2, \ldots , q_m\}$, where $q_k$ represents the query of candidate *k*-sequences in the *k*th iteration process. The sensitivity of $q_k$ can be calculated as follows: In the *k*th iteration process of the straight solution, after adding or deleting any one sequence, the upper bound of affected candidate *k*-sequences is $C_{l}^{k}$. Suppose the maximum cardinality of sequence is *l*, the maximum number of *k*-sequence patterns contained in this sequence is $C_{l}^{k}$. Let $T_k$ represent the candidate *k*-sequences generated in the *k*th recursive mining, the sensitivity of $q_k$ is $\Delta f_k = min(C_{l}^{k},T_k)$. Let $del_k$ represent the invalid candidate *k*-patterns pruned from $T_k$ based on privHashMap. After pruning the invalid patterns, the sensitivity of $q_k$ is $\Delta f_k = min(C_{l}^{k},T_k)-|del_k|$. After pruning these infrequent candidates, the magnitude of the noise is reduced.

**(4) The overall algorithm description.**


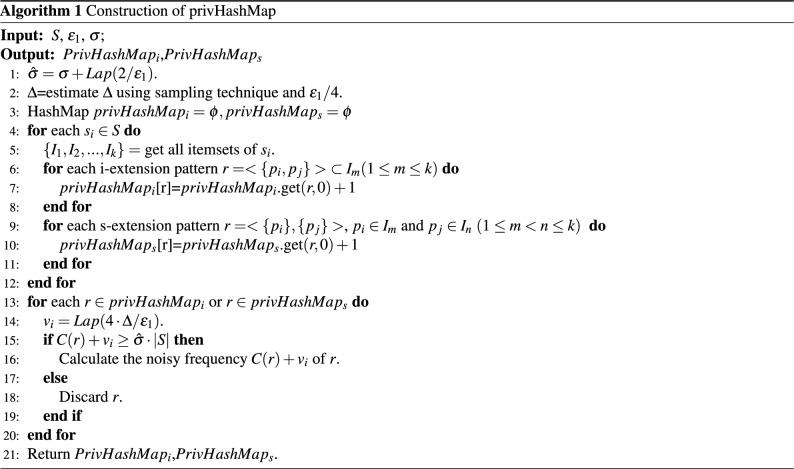



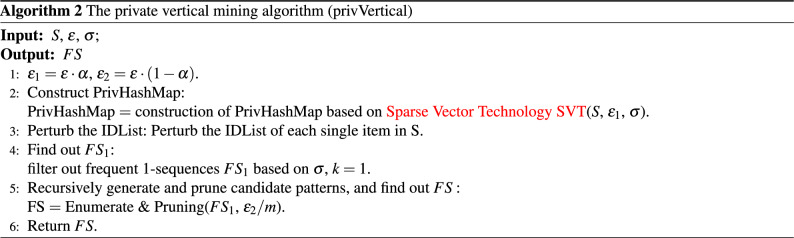



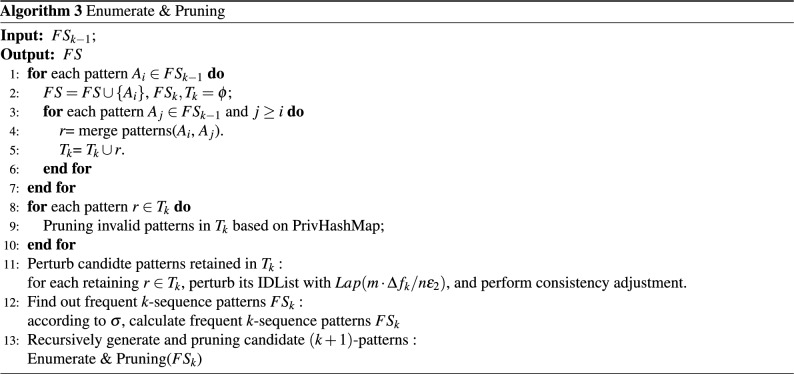


Algorithm 1 describes the construction of privHashMap. Firstly, perturb the true support threshold with $2/\varepsilon _1$(line 1), and estimate the number of frequent co-occurrence items $\Delta$ with $\varepsilon _1/4$ (line 2). Next, for each item $p_i$, find out s-extension items and i-extension items and calculate their co-occurrence frequency, then record them in $PrivHashMap_i$ and $PrivHashMap_s$ respectively (lines 3–12). Traverse *PrivHashMap* (line 13), for each *r* in $PrivHashMap_i$ and $PrivHashMap_s$, perturb its true support with $\varepsilon _1/4$ (line 14). If the noisy support is no less than the noisy support threshold, add retained it in PrivHashMap (lines 15–16). Otherwise, discard *r*. Then continue to traverse $PrivHashMap_i$ and $PrivHashMap_s$ until all elements in *PrivHashMap* have been traversed (lines 17–20). At last, return PrivHashMap (line 21).

Algorithm 2 describes the private vertical mining process: PrivVertical consists of three steps: the first is the construction of privHashMap, the second is the perturbation of IDList, and the third is generating and pruning candidate patterns based on the noisy $FS_1$. To make algorithm 2 satisfy $\varepsilon$-differential privacy, $\varepsilon$ is divided into two parts $\varepsilon _1$ and $\varepsilon _2$. $\varepsilon _1$ is used to construct the privHashMap (Algorithm 2: line 2). The remaining budget $\varepsilon _2$ is used for private vertical mining. Allocate $\varepsilon _2/m$ to each recursive process, construct noisyIDList of 1-sequences with $\varepsilon _2/m$, and find out $FS_1$ (Algorithm 2: lines 3–4). Then Recursively call the process of Enumerate & Pruning, generate and prune candidate patterns to get frequent *k*-patterns (Algorithm 2: lines 5–6).

Algorithm 3 describes the process of Enumerate & Pruning: (1) For any pattern $A_i\in FS_k$, add it to *FS* and output it (Algorithm 3: lines 1–2); (2) For each pattern $A_j\in FS_k$, merge $A_i$ and $A_j$ as *r* (Algorithm 3: lines 3–4) and add it to $T_k$ (Algorithm 3: lines 5–7). (3) Pruning invalid patterns in $T_k$ based on privhashmap (Algorithm 3: lines 8–10). (4) For each pattern *r* retained in the candidate set, construct its noisyIDList. (5) Calculate frequent *k*-sequence patterns $FS_k$ based on noisyIDList (Algorithm 3: line 12). (6) Let $FS_k$ as the input parameter, recursively call Algorithm 3 to mine frequent $(k + 1)$—sequence patterns (Algorithm 3: line 13).

### Complexity analysis

Algorithm 2 describes the overall scheme. It consists of Algorithm 1 and Algorithm 3. Let *N* represent the dataset size (the number of sequences). Let *max*|*s*| represent the max cardinality of the sequence, and $C_{max|s|}^2$ is the number of combinations of different co-occurrence items. Thus the complexity of Algorithm 1 is $O(N\times C_{max|s|}^2)$. Suppose the max length of frequent patterns is $p_{max}$, $|FS_K|$ denotes the number of frequent k-sequence patterns, and $C_{|FS_K|}^2$ is the extension number when generating candidate (k+1)-patterns based on $FS_K$. Let $T_{k+1}$ denote the candidate (k+1)-patterns. Thus the complexity of Algorithm 3 is $O(C_{|FS_K|}^2+T_{k+1})$, and the complexity of Algorithm 2 is $O(N\times C_{Max|s|}^2+p_{max}\times C_{|FS_K|}^2+T_{k+1})$. Algorithm 2 has low complexity. To reduce the complexity, Algorithm 2 constructs PrivHashMap to filter excessive candidate patterns. After reducing the size of candidates, the perturbation is also reduced. Thus the low complexity of Algorithm 2 can be ensured. Detailed running time evaluation can be seen in the “Efficiency evaluation” of experiments section.

## Privacy and utility analysis

### Privacy analysis

#### Theorem 4

The private vertical mining satisfies $\varepsilon _2$-differential privacy.

#### Proof

Suppose the maximum cardinality of frequent patterns is *m*, the private vertical mining process consists of *m* recursive sub-processes. Let $Q=\{q_1,q_2,\ldots ,q_m\}$ represent the query function in this process, where $q_k$ represents the query function of the *k*th recursive process. *FS* represents the frequent patterns set, and $FS_k$ represents the frequent *k*-sequence patterns set. $\Delta f_k$ represents the sensitivity of the *k*th recursive process. If we demonstrate that the following equation is true, we can get the private vertical mining satisfies $\varepsilon _2$-differential privacy.

4 $$\begin{aligned} e^{-\varepsilon _2} \le \frac{Pr[Q(S)\in FS]}{Pr[Q(S')\in FS]} \le e^{\varepsilon _2}\vspace{-0.3cm} \end{aligned}$$

A detailed analysis is as follows:

5 $$\begin{aligned} \begin{array}{ll} \frac{Pr[Q(S)\in FS]}{Pr[Q(S')\in FS]} &{}= \Pi _{k=1}^{m}\frac{exp(-\frac{\frac{n\varepsilon _2}{m}\cdot |q_k(S)|-|FS_k||}{\Delta f_k})}{exp(-\frac{\frac{n\varepsilon _2}{m}\cdot |q_k(S')|-|FS_k||}{\Delta f_k})} \\ {} &{}= \Pi _{k=1}^{m}exp(-\frac{\frac{n\varepsilon _2}{m}\cdot (|q_k(S')|-|FS_k||- (|q_k(S)|-|FS_k||))}{\Delta f_k}\\ {} &{}\le \Pi _{k=1}^{m}exp(-\frac{\frac{n\varepsilon _2}{m}\cdot (|q_k(S')|-|q_k(S|)}{\Delta f_k})\\ {} &{}\le exp(m \cdot \frac{\varepsilon _2}{m})\\ {} &{}= exp(\varepsilon _2) \end{array} \end{aligned}$$

In the above proof, the first inequality is inferred from the triangle inequality theorem, and the second inequality is inferred from the sensitivity definition ($|q_k(S')|-|q_k(S)|\le \Delta f_k$). Evidenced by the same token, the following equation holds:

6 $$\begin{aligned} \frac{Pr[Q(S)\in FS]}{Pr[Q(S')\in FS]}\ge exp(-\varepsilon _2) \end{aligned}$$

Therefore, the private vertical recursive mining algorithm satisfies $\varepsilon _2$-differential privacy.

#### Theorem 5

PrivVertical satisfies $\varepsilon$-differential privacy.

#### Proof

PrivVertical consists of two components: one is the private construction of PrivHashMap, the other is the private vertical mining. According to theorem 3, the the private construction of PrivHashMap satisfies $\varepsilon _1$-differential privacy. According to theorem 4, the private vertical mining satisfies $\varepsilon _2$-differential privacy. According to Theorem 1, PrivVertical satisfies $(\varepsilon _1+\varepsilon _2)$-differential privacy. Since $\varepsilon =\varepsilon _1+\varepsilon _2$, PrivVertical satisfies $\varepsilon$-differential privacy.

### Utility analysis

#### Theorem 2

For any $\beta >0$, at least with the probability of $1-\beta$, the upper-bound error between noisy frequency and true frequency of frequent *k*-patterns is $\gamma$, where $\gamma =O(\frac{m\cdot \Delta f_k}{n\varepsilon _2}ln\frac{\Delta f_k}{\beta })$.

#### Proof

Suppose *r* is a candidate *k*-sequence, and its true frequency is *c*(*r*). Since the perturbed noise to the true frequency is $Lap(m\cdot \Delta f_k/n\varepsilon _2)$, the probability that the error between true frequency and noisy frequency of *r* is no less than:

$$2\cdot \left( \frac{n\varepsilon _2}{2m\cdot \Delta f_k}\int _{c(r)+\gamma }^{+\infty }exp(-\frac{(x-c(r)n\varepsilon _2)}{m\cdot \Delta f_k})dx \right) = exp(\frac{-\gamma n\varepsilon _2}{m\cdot \Delta f_k})$$

In the *k*th recursive mining process, since the number of perturbed candidate *k*-patterns is $\Delta f_k$, the union upper bound of the probability less than $\gamma$ is $\Delta \cdot exp(\frac{-\gamma n\varepsilon _2}{m\cdot \Delta f_k})$, that is $\beta =\Delta \cdot exp(\frac{-\gamma n\varepsilon _2}{m\cdot \Delta f_k})$, and thus $\gamma =O(\frac{m\cdot \Delta f_k}{n\varepsilon _2}ln\frac{\Delta f_k}{\beta })$.

## Experimental results

We conduct experiments to evaluate the utility and efficiency of PrivVertical. All algorithms are implemented with Java.

### Comparison

Our experiments include the following comparison algorithms: (1) Prefix^8^: a representative privacy-preserving scheme based on perfixspan, which is implemented by perturbing the prefix tree. (2) PrivApriori: a representative private mining scheme based on an Apriori-based algorithm^9^. These two methods are representative horizontal mining algorithms with differential privacy. They are compared with PrivVertical, which is a vertical mining algorithm with differential privacy.

### Metrics

We adopt the following metrics to measure utility: F-score and RE^9^. *F*-score is used to measure the utility of the private mining results. The definition of F-Score is as follows:

7 $$\begin{aligned} F-score = 2\times \frac{precision\times recall}{precision + recall} \end{aligned}$$

where $precision=\frac{|\widehat{FS}\cap FS|}{|\widehat{FS}|}$,$recall=\frac{|\widehat{FS}\cap FS|}{|FS|}$. $\widehat{FS}$ is the noisy frequent sequence patterns of the private mining scheme, and *FS* is the original frequent sequence patterns of the no-private mining scheme.

RE (Relative Error) is used to measure the error between actual support and noisy support, which is defined as follows:

8 $$\begin{aligned} RE = AVG_{x\in FS}{\frac{sup_x^{'}-sup_x}{sup_x}} \end{aligned}$$

where $sup_x$ denotes the true support, and $sup_x^{'}$ denotes the noisy support.

Running time is used to measure the efficiency of algorithms.

### Datasets

Real datasets used in experiments are MSNBC and Kosaarak, which record the URL categories visited by users in time order, and click stream data respectively. Datasets can be obtained from the SPMF website. Detailed information can be seen in Table 1. |*S*| is the number of records of the dataset, |*I*| is the number of distinct items, and Max|*s*| and Avg|*s*| denote the maximal and the average record length respectively.

**Table 1:** Detailed information of datasets.

Dataset	$\left| S \right|$	|*I*|	Max|*s*|	Avg|*s*|
MSNBC	989,818	17	14,975	4.7
Kosaarak10k	10,000	10094	699	8.1

### Effect of $\varepsilon$ on utility

**Figure 5:**
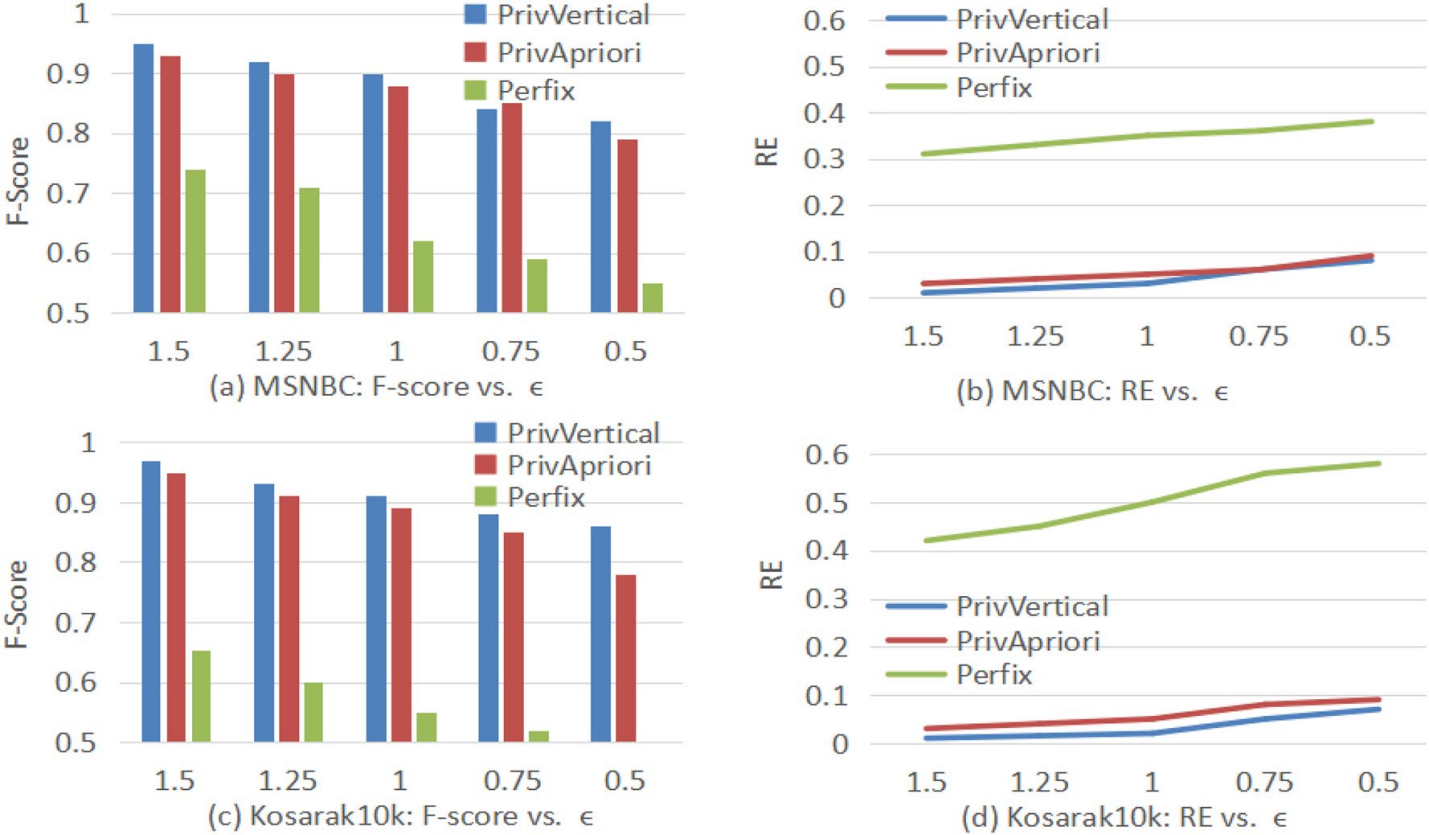
Effect of $\varepsilon$ on Utility.

Figure 5 shows how the parameter $\varepsilon$ affects the accuracy of the three algorithms.

In general, privVertical performs better under the same privacy level. The main reasons are as follows: PrivVertical prunes many invalid candidate patterns in private mining, the sensitivity is reduced and the noise required is also reduced. Although PrivApriori reduces the sensitivity by shrinking sequences and pruning invalid candidate patterns, there exists a sampling error in private mining. Prefix uses a prefix tree to reorganize the sequence database, and uses the projection technique to calculate the noisy support of a pattern. It contains the reorganization error, which results in low accuracy. As $\varepsilon$ increases, the privacy level decreases, F-Score increases, and RE decreases. The reason is as follows: the higher the parameter $\varepsilon$, the lower the privacy level is. A lower privacy level means the required noise is lesser, improving the accuracy. Compared with MSNBC, the utility of Kosarak is higher. The reason may be that the candidate patterns on kosarak are sparse, the number of candidate patterns is relatively small, and the pruning strategy is more effective.

### Effect of pruning strategy on utility

We evaluate how effective of the pruning strategy based on privHashMap on utility in this section.

**Figure 6:**
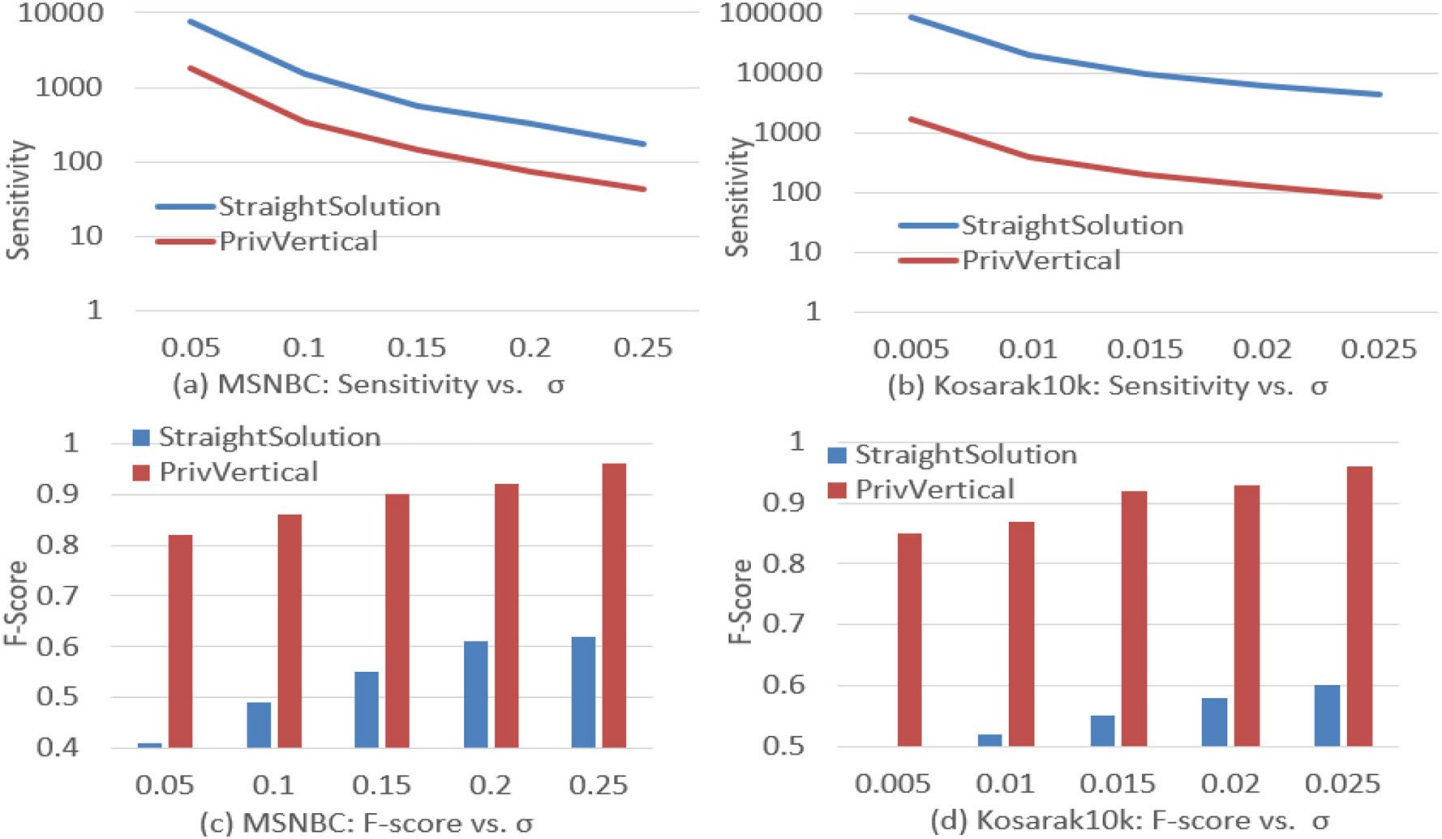
Effect of *pruning* based on privHashMap on utility.

The private vertical mining scheme without candidate patterns pruning is named as StraightSolution. From Fig. 6a, b, we can see the sensitivity reduction rate on MSNBC in the private mining achieves to 74–78%. The reduction rate on Kosarak achieves to 98%. From Fig. 6c, d, Compared with StraightSolution, privVertical can greatly improve the utility. The main reason is that the sensitivity is reduced, the noise required decreases.

### Efficiency evaluation

**Figure 7:**
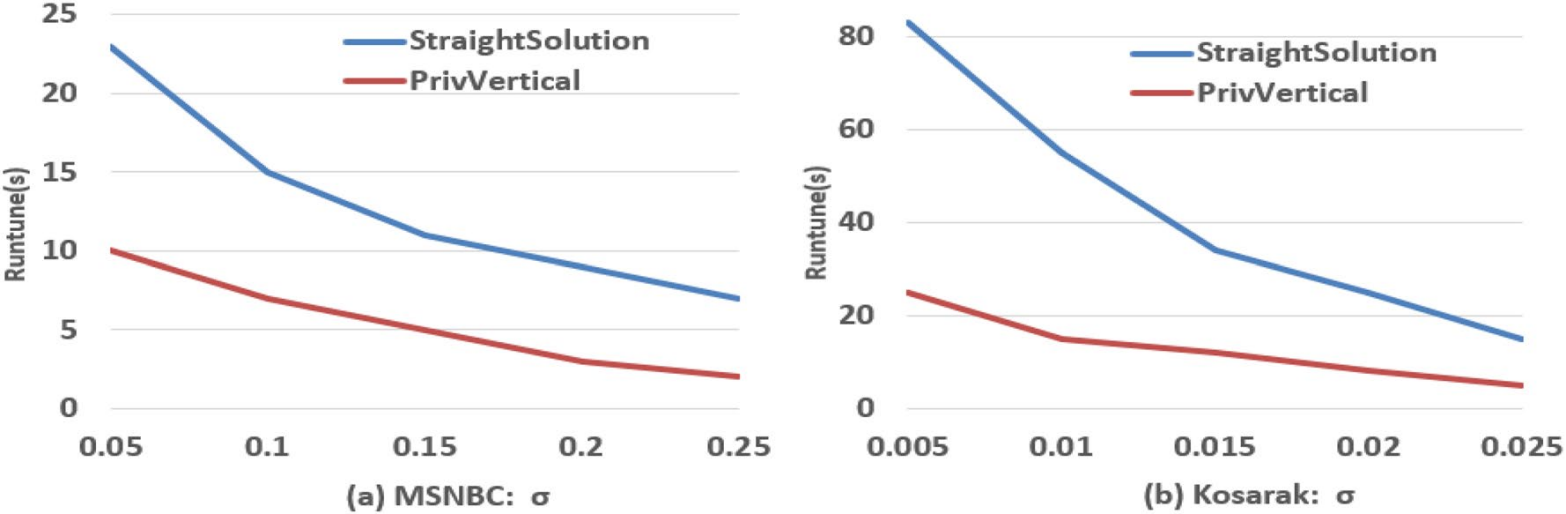
Effect of pruning based on privHashMap on efficiency.

Figure 7 shows how the pruning strategy based on privHashMap affects efficiency. We can see PrivVertical performs better than the StraightSloution. After pruning invalid candidate patterns, the size of candidate patterns set is lower. In this way, it will take less time to make the private processing for the candidate patterns, thus the running time is reduced. With the increase of $\sigma$, the efficiency of the two schemes is decreasing, this is because the candidate patterns space becomes smaller.

## Conclusion

In this study, we analyzed why existing works can not afford a private sequence patterns mining scheme with a high level of privacy while achieving both high utility and efficiency. The first reason is the low efficiency of the horizontal mining style. The second reason is low utility caused by too many candidate patterns generated in private mining. To address the above issue, privVertical, a private vertical sequential pattern mining scheme is proposed for the first time. High efficiency is attained by reducing the scanning times of database in the private mining process. It is implemented by perturbing a non-private vertical mining algorithm with differential privacy. The utility is enhanced by less noise required for the same level of privacy. It is implemented by two strategies: The first is designing the privHashMap. It is a private co-occurrence hash map list designed based on the Sparse Vector Technology and used to filter invalid candidate patterns in the subsequent private mining process. Therefore the magnitude of noise required for privacy protection can be reduced. Secondly, a noise addition scheme for the vertical mining algorithm is designed, which can improve the mining efficiency while satisfying differential privacy constraints.

By formal theoretical analysis, the upper bound of the utility of privVertical is given, and the privacy bound of privVertical is also proved. Compared with other state-of-art methods, experiments verified that privVertical has higher accuracy and efficiency under the same privacy budget. This is because the Laplace noise required for perturbing the support of candidate patterns is reduced greatly. In the experiments, the candidate reduction rate on two real datasets achieves 74–98%, which greatly improves the utility of private mining. Our experimental results also demonstrated how the pruning strategy based on privHashMap affects efficiency. After pruning invalid candidate patterns, the size of the candidate patterns set is lower. In this way, it takes less time to make the private processing for the candidate patterns, and thus the running time is reduced. In future work, PrivVertical will be applied to other practical problems, such as product recommendation and biomedical data analysis. Another possible future work may also include testing the proposal using other practical datasets or metrics related to quality.

## Data Availability

The current research is available from the corresponding author upon reasonable request, and the data used and/or analyzed during the current study available from http://www.philippe-fournier-viger.com/spmf/index.php?link=datasets.php online.
